# Comprehensive Transcriptomic Analysis and Experimental Validation of Notochordal Cells and Nucleus Pulposus Cells: Uncovering Novel Therapeutic Targets for Intervertebral Disc Degeneration

**DOI:** 10.3390/cimb47121001

**Published:** 2025-11-28

**Authors:** Yanhu Li, Peng Cheng, Haijun Zhang, Shijie Chen, Huan Liu, Kun Wang, Jing Wang, Xuewen Kang

**Affiliations:** 1 The Second Hospital & Clinical Medical School, Lanzhou University, 82 Cuiyingmen, Lanzhou 730030, China; 220220904261@lzu.edu.cn (Y.L.); chengp20@lzu.edu.cn (P.C.); zhjmoon@163.com (H.Z.); chenshj2023@lzu.edu.cn (S.C.); liuh2024@lzu.edu.cn (H.L.); wangk2024@lzu.edu.cn (K.W.); wjzgrwj@163.com (J.W.); 2 Orthopaedics Key Laboratory of Gansu Province, Lanzhou 730030, China

**Keywords:** intervertebral disc degeneration (IDD), notochordal cells (NCs), nucleus pulposus (NP), transcriptomic analysis, SOD2, CAT

## Abstract

Current therapeutic strategies for intervertebral disc degeneration (IDD)-related low back pain are limited to symptomatic alleviation. Notochordal cells (NCs), as progenitor cells of the nucleus pulposus (NP), lead us to develop innovative NC-based new therapies for IDD. A total of 40 NP specimens, obtained according to IDD criteria with defined Pfirrmann grades and histological degeneration score, were categorized as either normal (Grade II) or degenerated (Grade IV). An IDD model was established in SD rats by needle puncture of the annulus fibrosus. Degenerated NP tissue was identified using MRI, H&E, Safranin O, and Masson staining. NCs and NP cells (NPCs) were isolated and identified based on specific cellular markers. Furthermore, mRNA-seq was performed to profile gene expression in these cells. GO annotation and KEGG pathway analysis were employed to perform functional enrichment analysis of the differentially expressed genes (DEGs). Cell viability was assessed using the CCK-8 assay. An in vitro cell degeneration model was established by treating NPCs with TBHP. Analysis of specific marker expression was performed using Western blotting, immunohistochemistry, and immunofluorescence. We found that the number of NCs in degenerated NP tissues was significantly reduced compared to those in normal NP tissues, but a small amount of notochordal cell markers could still be detected. Analysis of sequencing data identified 2391 upregulated and 3813 downregulated DEGs. GO enrichment analysis indicated that these DEGs were significantly associated with regulatory signals including cellular senescence and oxidative stress. KEGG pathway analysis further revealed that the DEGs were primarily enriched in the TNF and HIF-1 signaling pathways. Subsequent screening identified the top 10 key genes potentially related to IDD: *Sod2*, *Cxcl12*, *Spp1*, *Fn1*, *Cat*, *Il6*, *Ccl2*, *Igf1*, *Fgf2*, and *Acta2*. Collectively, our findings establish a clear link between SOD2/CAT and the pathogenesis of IDD. SOD2 and CAT may serve as promising new potential therapeutic targets for IDD by inhibiting oxidative stress and cellular senescence in NPCs.

## 1. Introduction

The intervertebral disc (IVD) is a fibrocartilaginous articular structure consisting of the central NP, the peripheral annulus fibrosus (AF), and the superior and inferior cartilaginous endplates (CEPs). It acts as a shock absorber during spinal movements such as flexion, extension, and rotation [1]. The NP is composed of NPCs and the extracellular matrix (ECM) they secrete, plays a crucial role in resisting axial loads of the spine, and is therefore essential for the proper functioning of the IVD [2]. In the NP, activation of antioxidant mechanisms is crucial for counteracting external stimuli, which may originate from mechanical stress, inflammatory responses, or other biological factors. When confronted with these challenges, NPCs upregulate the expression of antioxidant enzymes to protect against oxidative damage, thereby preserving cellular proliferation and metabolic functions. Furthermore, NPCs participate in cell signaling by modulating the expression of ECM components. This signaling not only influences cell growth and survival but also plays a pivotal role in maintaining the overall function and structural integrity of the NP. Alterations in the ECM can impact the interaction between NPCs and their microenvironment, thereby shaping responses to injury and repair processes.

IDD is an age-related degenerative spinal disease caused by dysfunction of the NP, leading to significant impairment in both work ability and quality of life [3,4,5]. IDD can lead to a variety of spinal disorders manifesting as acute or chronic low back pain (LBP), including conditions such as disc herniation, spondylolisthesis, and spinal stenosis [6,7]. The global prevalence of LBP is 7.3% and affects approximately 540 million people worldwide [8]. A report on the global burden of disease identified LBP as a leading cause of disability [9,10,11]. Currently, the treatment of LBP mainly includes conventional therapies such as physiotherapy, injection treatments and surgery, as well as pharmacological treatments with prescription opioids, non-steroidal anti-inflammatory drugs (NSAIDs) and muscle relaxants [12]. However, these treatments have limitations as they are primarily aimed at relieving symptoms and controlling inflammation rather than fundamentally reversing the progression of IDD. Therefore, it is crucial to further investigate the mechanisms underlying IDD and to develop novel therapies that target these mechanisms.

In recent years, stem cell therapy has gained significant attention due to its potential to treat early IDD while preserving the mechanical properties of the spine. Numerous clinical trials have investigated the use of allogeneic mesenchymal stem cells (MSCs) for the treatment of IDD. These studies have shown promising results in improving symptoms and pain relief, demonstrating the therapy’s potential for improved outcomes [13,14]. In particular, NCs, which occur in IVD as nucleus pulposus progenitor cells (NPPCs) or nucleus pulposus progenitor cells, also possess stem cell-like properties as they can directly differentiate into NPCs. Therefore, these NCs are expected to offer better prospects for IDD treatment compared to other MSCs [15,16]. However, research into using NCs to treat IDD is still in its infancy. Against this background, the development of new cell therapies for IDD based on NCs represents both a challenging and exciting opportunity with significant long-term potential.

NCs arise from embryonic notochordal cells (eNCs) during fetal development and are characterized by the presence of different vacuoles [12,17]. Recent studies suggest that the number of NCs decreases starting in the second decade of an individual’s life, which coincidentally coincides with the onset of early IDD [18,19,20]. This highlights the critical role that NCs play in the development and function of IVD. Although some studies suggest that NCs can activate NPCs [21] and mesenchymal stem cells (MSCs) [22], paving the way for NC-based IDD treatments, it is crucial to first determine whether NCs in adult or degenerated NP tissues. The loss of NCs in humans is recognized as a hallmark feature of IDD. To investigate the underlying mechanisms, functional studies are essential; however, the acquisition of viable human NCs remains unfeasible. Therefore, we turned to the well-established rat model, which is rich in NCs and widely regarded as a gold standard for studying NC biology.

To further explore NC-based therapies for IDD, we isolated NCs and NPCs from rats and performed mRNA sequencing (mRNA-seq). mRNA-seq, a high-throughput sequencing technology (also known as next-generation sequencing, NGS), enables comprehensive analysis of all mRNA transcribed from tissues or cells under specific functional conditions. This method enables the precise identification of coding SNPs (cSNPs) [23,24], alternative splicing events (AS) [25,26], and other transcriptomic features and offers unique advantages in detecting low-abundance transcripts and discovering new transcript variants. Because of these capabilities, mRNA sequencing is widely used in basic research, clinical diagnostics, and drug development.

The conceptual framework of our study is built upon the following logical progression: First, we posit that NCs possess protective properties against IDD. Therefore, we identified the core molecular characteristics distinguishing NCs from their descendant NPCs and then leveraged public transcriptomic datasets to demonstrate the correlation between these key molecules and IDD. We then reasoned that the depletion of these NC-enriched protective molecules in degenerated tissues may actively contribute to IDD pathogenesis. Finally, we experimentally validated this premise by demonstrating a significant inverse correlation between the expression levels of these specific molecules and degenerative status in both human clinical specimens and established disease models. In essence, our study compares NCs (with their regenerative potential) to their NPCs descendants to define the unique protective molecular library of NCs, ultimately seeking to determine if the depletion of its critical components contributes to IDD.

In this study, we are the first to demonstrate the association between NCs and IDD using human surgical samples and the AF puncture-induced rat model of IDD. We performed a series of bioinformatics analyzes of mRNA-seq data from rat NCs and NPCs and identified two potential molecular targets—SOD2 and CAT—that may play a key role in IDD progression. Finally, we validated these results in both human and rat nucleus pulposus cell lines and demonstrated that these targets mediate IDD by regulating oxidative stress in NPCs. Our results provide new insights into the role of NCs and may inform future studies on the molecular mechanisms underlying the involvement of NCs in IDD. Furthermore, these results provide valuable data for the development of NC-based targeted therapies for IDD.

## 2. Materials and Methods

### 2.1. Human NP Tissue Collection

The experimental protocol involved in this study was approved by the Ethics Committee of The Second Hospital & Clinical Medical School, Lanzhou University (Approval Code: 2024 A-258; Approval Date: 30 March 2024). Procedures involving human subjects follow the guidelines of the Declaration of Helsinki and obtain disc nucleus pulpous tissues with the written informed consent of all patients. Preoperative T2-weighted magnetic resonance imaging (MRI) and image analysis were performed by 3 independent observers and Pfirrmann grading was performed [27]. NP tissues were harvested from thoracic discs excised for spinal correction during scoliosis surgery. Although the spines were deformed, these Pfirrmann grade II discs from young patients exhibited intact ECM and high cellular viability upon histological and MRI evaluation. Therefore, they have been widely adopted in the literature as relatively normal controls. The normal samples (Grade II) were obtained from adolescent patients (9 males, 10 females; Age 12–17 years, mean 14.6 years) with idiopathic scoliosis, while the degenerative samples (Grade IV) were collected from patients (11 males, 10 females; Aged 52–75 years, average 65.3 years) who underwent surgery for disc herniation or disc fusion. Patients with congenital or syndromic scoliosis were excluded from the study. Immediately after the surgery, the nucleus pulposus samples were immersed in 4% formaldehyde solution for preservation to maintain the structure and cell integrity. Throughout the sample collection and processing, strict protocols were followed, and all samples were anonymous to ensure the privacy and confidentiality of the patients. The management of the collected samples complies with ethical norms, legal authorizations, and regulatory requirements. All patients underwent surgical treatment in The Second Hospital & Clinical Medical School, Lanzhou University. Patients with history of infectious diseases such as tumor and tuberculosis were excluded before operation.

### 2.2. Hematoxylin-Eosin (H&E) Staining, Safranin O Staining and Masson Staining

To assess the extent of IDD in human SD rats, 5 μm thick sections were prepared after tissue fixation, decalcification, dehydration, and paraffin embedding (human disc specimens were free of decalcification). H&E staining was performed using the H&E dyeing kit (Solarbio, Beijing, China) according to the manufacturer’s instructions. In addition, to enhance the analysis of cartilage and disc tissue integrity, we added modified Safranin O staining and Masson staining, and based on the modified Safranin O staining kit (Solarbio, Beijing, China) and Masson staining kit (Solarbio, Beijing, China) instructions for dyeing. The stained sections were carefully examined and recorded using an optical microscope (Olympus, Tokyo, Japan) for a comprehensive understanding of the structural changes associated with IDD.

### 2.3. Histological Grading of Intervertebral Disc Degeneration

To objectively evaluate the extent of intervertebral disc degeneration, histological scoring was performed on all human and rat disc tissue sections.

The scoring criteria were as follows:

Sections stained with H&E were evaluated based on four categories:

NP Cell Morphology:
ScoreScoring criteria1 pointPredominance of notochordal cells; cells are large with vacuolated cytoplasm2 pointsMixed population of notochordal cells and chondrocyte-like cells3 pointsExclusively chondrocyte-like cells; cells are small and round, resembling chondrocytes4 pointsMarked hypocellularity with evidence of necrosis or apoptosis

Demarcation Between NP and AF:
ScoreScoring criteria1 pointClear and well-defined border2 pointsMildly irregular border3 pointsModerately irregular border with beginning protrusion of NP into the AF4 pointsComplete loss of border; NP and AF tissues are intermingled

AF Structure:
ScoreScoring criteria1 pointCollagen lamellae are distinct and well-organized without disruption2 pointsSlight waviness or mild disorganization of lamellar structure3 pointsModerate disorganization with localized separation or breaks4 pointsSevere disruption of lamellar structure with fissures or tears

Granulation Tissue Formation:
ScoreScoring criteria1 pointNo vascularized granulation tissue infiltration2 pointsMild vascular infiltration in the outer AF3 pointsVascular infiltration extending into the inner AF4 pointsVascularized granulation tissue invading the NP region

Scoring Procedure:

Scores from the four categories were summed to yield a total score for each sample (range: 4–16), with higher scores indicating more severe degeneration. All sections were evaluated independently by three experienced researchers blinded to sample group assignment. The final score for each sample was calculated as the mean of the three observers’ scores. Statistical analysis confirmed good inter-observer agreement (intraclass correlation coefficient, ICC > 0.85).

### 2.4. Protein Isolation from NP Tissue

NP tissues from human and SD rats were mixed with a pre-cooled 0.1 M ammonium acetate-methanol solution at a ratio of 5:1 and precipitated overnight at −20 °C. The sediment was collected by centrifugation at 4 °C and 12,000× *g* for 10 min. Then, the pre-cooled methanol was washed twice at a ratio of 5:1, gently mixed, and centrifuged at 12,000× *g* to collect the precipitation. The residual methanol is removed with acetone. After the dry precipitate was dissolved in the sample lysate at room temperature for 3 h, the solution was centrifuged twice at room temperature at 12,000× *g* and reacted for 10 min to obtain the supernatant. The supernatant contains the total protein content of the sample, quantified using a BCA protein detection kit (Thermo Fisher Scientific, Waltham, MA, USA) and stored at −80 °C.

### 2.5. Western Blotting

RIPA lysis buffer (Beyotime, Shanghai, China) is used to extract proteins from NP tissues and NPCs. The protein concentration was determined using the Bradford Protein Assay kit (Thermo Fisher Scientific). The same amount of protein was isolated by 10% sodium dodecyl sulphate-polyacrylamide gel electrophoresis (SDS-PAGE) and transferred to a polyvinylidene fluoride (PVDF) membrane (Millipore, Billerica, MA, USA). It was sealed with Quickblock™ blotting solution at room temperature for 15 min and then overnight at the corresponding antibody concentration at 4 °C. The membrane was then washed with TBST (Beyotime, Shanghai, China) 3 times for 5 min each time and then with either a goat anti-rabbit antibody coupled with horseradish peroxidase (1:500, Proteintech, Wuhan, China) or a goat anti-mouse antibody coupled with horseradish peroxidase (1: 5000, Proteintech, Wuhan, China) incubated at room temperature for 1 h. Protein signals were detected using hypersensitive luminescent solution (New Cell Molecular Biotech, Suzhou, China) and ChemiDoc XRS Imaging System (Bio-Rad, Hercules, CA, USA). The protein band density was quantified using the ImageJ software (version 1.48) and the result was expressed as the relative level of β-actin.

Anti-Cytokeratin 18 (CK18) (Abmart, Shanghai, China), 1:1000; Anti-Brachyury (Abmart, Shanghai, China), 1:1000; Anti-Vimentin (Abmart, Shanghai, China), 1:1000; Anti-SOD2 (Proteintech, Wuhan, China), 1:1000; Anti-CAT (Proteintech, Wuhan, China), 1:1000; β-actin (Proteintech, Wuhan, China),1:2000; Anti-P16 (Cell Signaling Technology, Danvers, MA, USA), 1:1000; Anti-P21 (Cell Signaling Technology, Danvers, MA, USA), 1:1000; Anti-p-P53 (Cell Signaling Technology, Danvers, MA, USA), 1:1000; Anti-mouse secondary antibody (Proteintech, Wuhan, China), 1:5000; Anti-rabbit secondary antibody (Proteintech, Wuhan, China), 1:5000.

### 2.6. Immunohistochemical (IHC) Staining

Specimens were sliced from human and rat intervertebral discs. After tissue fixation, decalcification, dehydration, and embedding, sections 5 μm thick were prepared (human disc specimens were exempt from decalcification). After antigen repair and endogenous enzyme clearance, the slices were incubated overnight with primary antibodies (CK 18, Brachyury, Vimentin, SOD2 and CAT) at 4 °C. These slices were then combined with goat anti-rabbit or anti-mouse immunoglobulin (Ig) G-horseradish peroxidase HRP (1:500; ZSGB Bio, Beijing, China) at 37 °C for 30 min. Finally, 3′,3-diaminobenzidine tetra methylene hydrochloride solution was added, and the nuclei were stained with hematoxylin and observed under optical microscope (Olympus, Tokyo, Japan).

Anti-Cytokeratin 18 (CK18) (Abmart, Shanghai, China), 1:50; Anti-Brachyury (Abmart, Shanghai, China), 1:50; Anti-Vimentin (Abmart, Shanghai, China), 1:50; Anti-SOD2 (Proteintech, Wuhan, China), 1:50; Anti-CAT (Proteintech, Wuhan, China), 1:50.

### 2.7. Rat Model of IDD

Previous studies have shown that AF puncture-induced IDD rat models are mature IDD models [28]. In this study, SD rats weighing 200 to 220 g were used. The rats were obtained from the Gansu Provincial Animal Science Center. Anesthesia was induced with pentobarbital sodium at a dose of 45 mg/kg. IDD rat models were created by surgical intervention under sterile conditions. The anesthetized SD rats were divided into two groups: control group and AF group. The postural position was prone and the midline longitudinal incision was made along the tail. The procedure involves removing the left facet joint between the third and fourth lumbar vertebrae to visualize the Co5/6 disc. A 30-gauge needle was then carefully inserted parallel to the endplate, inserted 3.0 mm into the disc and held in place for 30 s. No intervention was performed in the control group. The muscle layer was sutured with 3-0 silk thread, while the skin was sutured with 4-0 nylon suture. SD rats were killed 8 weeks after the operation for follow-up experiments. All procedures were reviewed and approved by the Ethics Committee of The Second Hospital & Clinical Medical School, Lanzhou University (Approval Code: D2024-303; Approval Date: 30 March 2024). The study adhered to the ethical standards outlined in the Laboratory Animal Care and Use Guide (NIH, 2011), the Basel Declaration, and the Helsinki Declaration of 1964, ensuring scientific and ethical integrity.

### 2.8. Magnetic Resonance Imaging (MRI)

To evaluate the extent of IDD induced by annular puncture in Sprague-Dawley (SD) rats, in vivo magnetic resonance imaging (MRI) of the rat tails was performed at 8 weeks post-surgery using a 3.0 T MR scanner (Siemens, Erlangen, Germany). T2-weighted MRI sequences are particularly advantageous for monitoring the water content of nucleus pulposus (NP) tissue. Sequential T2-weighted sagittal images were acquired with the following parameters: a turbo spin-echo sequence with a repetition time (TR) of 5400 ms, an echo time (TE) of 920 ms, a matrix size of 320 (h) × 256 (v), a field of view (FOV) of 260°, and 4 excitations (NEX). The slice thickness was set at 2 mm with no gap. Intervertebral disc degeneration in the rat caudal spines was assessed according to the classification system described by Pfirrmann et al. [27].

### 2.9. Cultivation and Treatment of Human NPCs

Human NPCs was purchased from Shanghai Yaji Biotechnology Co., Ltd. (article number: 0028a, Shanghai, China). It was cultured in Dulbecco Modified Eagle medium/nutrient mixture F-12 (DMEM/F12) medium (Gibco, Thermo Fisher Scientific, Waltham, MA, USA) containing 10% fetal bovine serum (Biosharp, Hefei, China). Placed in a cell incubator containing 5% CO_2_ at 37 °C. Medium 2–3 times a week. Under an optical microscope, when the cell density reached 80–90%, the cells were digested with 0.25% trypsin ethylenediamine tetra acetic acid (Gibco, Thermo Fisher Scientific, Waltham, MA, USA) and passed in a 1:2 ratio. NPCs were added to the 6-well plate at a density of 1 × 10^5^ cells/well. Treated with TBHP (or without pretreatment) and cultured for 6 h, then used for subsequent immunofluorescence experiments.

### 2.10. Culture and Treatment of NPCs in SD Rats

Gel-like NP tissue was harvested from the tail of Sprague-Dawley (SD) rats at 3 to 4 weeks of age. These tissues were subjected to a 4-h digestive process using 0.25% type II collagenase (Millipore Sigma, Burlington, MA, USA) at 37 °C. After digestion, the tissue is rinsed with phosphate-buffered saline (PBS), It was then cultured in a Dulbecco Modified Eagle medium/nutrient mixture F-12 (DMEM/F12) medium (Thermo Fisher Scientific, Waltham, MA, USA) enriched with 15% fetal bovine serum (FBS) and 1% streptomycin/penicillin at 37 °C and 5% CO_2_. Once confluent, the cells were isolated using 0.25% trypsin-EDTA (Thermo Fisher Scientific, Waltham, MA, USA) and inoculated into a 10 cm dish at a specified density for subsequent proliferation. Under an optical microscope, when the cell density reached 80–90%, the cells were digested with 0.25% trypsin ethylenediamine tetra acetic acid (Gibco, Thermo Fisher Scientific, Waltham, MA, USA) and passed in a 1:2 ratio. NPCs were added to the 6-well plate at a density of 1 × 10^5^ cells/well. Treated with TBHP (or without pretreatment) and cultured for 6 h, then used for subsequent immunofluorescence experiments.

### 2.11. Extraction and Culture of NCs

Thirty-six adult male Sprague-Dawley rats weighing 200–220 g were purchased from the Laboratory Animal Center of Lanzhou University. All animal operations were performed under the approval and guidance of the Ethics Committee of the Second Hospital of Lanzhou University. Extract NCs according to the method introduced by Joo Han Kim et al. [29]. In short, after intravenous general anesthesia, the rats were killed and lumbar NP tissues were harvested from each disc. The NP tissues were then washed with phosphate-buffered saline (PBS) and digested in 0.2% Streptomycin (Gibco-BRL, Carlsbad, CA, USA) (0.005 mL/mg of tissue) for 40 min. After washing twice, the tissues were then placed in 0.25% type II collagenase (Gibco-BRL, Carlsbad, CA, USA) (0.002 mL/mg tissue) and stirred at 37 °C for 4 h. The tissue fragments were then separated by a nylon mesh with a 70 μm aperture. It has been shown that NCs do not adhere to the culture vial until day 6; Thus, these cells separated from chondrocyte-like NPCs on day 3. The NCs were then separated by a continuous filtration procedure with pore sizes of 40, 25, and 15 μm. The NCs produced were about 100 cells/mg tissue and the NPCs were about 200 cells/mg tissue. The NCs was inoculated in a culture vial containing a DMEM/F12-based medium containing 10% fetal bovine serum (Thermo Fisher Scientific, Waltham, MA, USA). The flask was then cultured in an incubator at 37 °C with 20% oxygen and 5% CO_2_.

### 2.12. Immunofluorescence (IF) Staining

Tissue immunofluorescence staining. Specimens were sliced from a rat disc. After tissue fixation, decalcification, dehydration and embedding, 5 μm thick slices were prepared. After antigen repair and endogenous enzyme clearance, they were blocked in PBS with 1% BSA for 1 h. The cells were then incubated at 4 °C for primary antibody overnight. After thorough washing, they were further incubated with anti-rabbit or anti-mouse Alexa Fluor-488 and Alexa Fluor-594 coupled secondary antibodies (from Invitrogen, Carlsbad, CA, USA) at room temperature at 1: Incubate at a ratio of 300 dilution for one hour. Then, 4′,6-diaminidine-2-phenylindole (DAPI, Invitrogen) was added to label the nucleus for 10 min. The final imaging step is performed using a laser scanning confocal microscope equipped with an LCPlanFl objective lens (manufactured by Olympus in Tokyo, Japan).

Cell immunofluorescence staining. The slide is placed in a 6-well plate and NPCs and NCs are inoculated onto it. After the corresponding treatment, the medium was removed and the cells were washed with pre-cooled PBS 3 times for 3 min each time. The cells were fixed with 4% paraformaldehyde for 15 min, and then washed 3 times with PBS for 3 min each time. Next, the cells were permeated with 0.5% Triton X-100 at room temperature for 20 min and the slides were washed with PBS three times for 3 min each time. It was closed with 10% goat serum at room temperature for 1 h and incubated with primary antibody at 4 °C overnight. The cells were then washed 3 times with PBS for 3 min each time and incubated with Alexa Fluor 488/594-goat anti-rabbit lgG at 37 °C for 1 h. Finally, 4′,6-diaminidine-2-phenylindole (DAPI, Invitrogen, CA) was added to label the nucleus for 10 min. Images were captured using a fluorescence microscope (Olympus, Tokyo, Japan). The ImageJ software is used to measure fluorescence intensity.

Anti-Cytokeratin 18 (CK18) (Abmart, Shanghai, China), 1:200; Anti-Brachyury (Abmart, Shanghai, China), 1:200; Anti-Vimentin (Abmart, Shanghai, China), 1:200; Anti-SOD2 (Proteintech, Wuhan, China), 1:200; Anti-CAT (Proteintech, Wuhan, China), 1:200.

### 2.13. The Sequencing Method and Analysis

For all samples included in the analysis, the mapping rates were greater than 90%, and ribosomal RNA-derived reads constituted less than 5% of the total reads. The lack of substantial batch effects was further verified by principal component analysis (PCA). The RIN values of all RNA samples used for sequencing were all 10 (Supplementary Table S1).

The raw sequencing data was obtained using the Illumina NovaSeq 6000 platform with a sequencing mode of PE150 (Pair-end 150 bp), meaning each end of the paired-end sequencing reads 150 bp. After obtaining the raw image files, base calling and error filtering were performed to generate the raw sequencing fragments, which we refer to as Reads. The quality control of the raw data was performed using FastQC (v0.11.9), and adapter trimming and low-quality base filtering were carried out using Trimmomatic (v0.39) with the following parameters: SLIDINGWINDOW:4:20, MINLEN:50. Data filtering was further applied using Seqtk, with the following steps: 1. Removal of adapter sequences within the reads; 2. Removal of bases with a quality score (Q) lower than 20 at the 3′ end, corresponding to an error rate lower than 0.01 (where Q = −10 × log(error ratio)); 3. Removal of reads shorter than 25 bases; 4. Removal of ribosomal RNA reads belonging to the species. The pre-processed reads were then mapped to the genome using the spliced mapping algorithm in Hisat2 (version: 2.0.4).

To ensure the comparability of gene expression levels across different genes and samples, the reads were converted to FPKM (Fragments Per Kilobase of exon model per Million mapped reads) for gene expression normalization. First, we used String tie (version: 1.3.0) to count the fragments for each gene after Hisat2 alignment. Then, we normalized the data using the TMM (Trimmed Mean of M-values) method. Finally, a Perl script was used to calculate the FPKM value for each gene.

Differentially expressed genes were selected based on a Q-value ≤ 0.05 and |log2FC| ≥ 1. Differential gene expression analysis between samples was performed using edge R, and *p*-values were obtained. Multiple hypothesis testing was then conducted with correction for False Discovery Rate (FDR) to determine the threshold for *p*-value, and the corrected *p*-value was referred to as the Q-value. Additionally, differential expression fold changes were calculated based on FPKM values.

### 2.14. The mRNA-Seq Data Analysis

The differentially expressed gene set (DEGs, *n* = 6204, including 2391 upregulated genes and 3813 downregulated genes) was functionally annotated using clusterProfiler (v4.0.5). GO biological process enrichment and KEGG pathway analysis were performed using the hypergeometric test, with a significance threshold set at FDR-adjusted *p*-value ≤ 0.05.

The specific principle of GO enrichment analysis is to map the selected differentially expressed genes to the various entries (terms) in the GO database, calculate the number of genes in each entry, and then apply the hypergeometric test to select the GO terms that are significantly enriched in the differentially expressed genes compared to the entire genomic background. The computed *p*-values are corrected for multiple hypothesis testing, and GO terms with q-value ≤ 0.05 are considered significantly enriched in the differentially expressed genes. To investigate the functions of DEGs, we first performed GO analysis for both upregulated and downregulated DEGs. The number of DEGs in each category was then quantified using three GO terms: biological process, cellular component, and molecular function. The results are presented as bar graphs. We then performed GO enrichment analysis and displayed the top 30 GO terms sorted by rich factor as scatterplots.

Additionally, KEGG pathway enrichment analysis of the differential genes can be performed using the same principle as for GO enrichment analysis. To further investigate the functions of the DEGs, we performed KEGG enrichment analysis. Similarly to GO analysis, we quantified the number of DEGs in each pathway and visualized the results using bar graphs. Furthermore, we presented the top 30 KEGG pathways organized by enrichment level.

### 2.15. Bioinformatics Validation of SOD2 and CAT and Creation of Violin Plots

Gene expression data of SOD2 and CAT were obtained from a publicly available transcriptomic dataset (GSE147383) comprising normal and IDD human nucleus pulposus samples. Differential expression analysis was performed using the R package DESeq2 (version 1.34.0), and normalized expression values (TPM) for SOD2 and CAT were extracted for visualization.

Violin plots were generated using the ggplot2 package (version 3.5.2) in R (version 4.3.0). The plots depict the distribution of SOD2 and CAT expression in normal versus IDD groups, with the width of the violin shape representing the density of data points. A boxplot is embedded inside each violin, displaying the median, quartiles, and potential outliers. Statistical significance between groups was assessed using the Wilcoxon rank-sum test (Mann–Whitney U test), and the resulting *p*-value is indicated above the groups.

All statistical analyses and visualizations were conducted in RStudio (version 2025.05.0), and a *p*-value of less than 0.05 was considered statistically significant.

### 2.16. Cell Viability Assay

Cell viability was assessed with CCK8 kit (BS350B, Biosharp, Hefei, China). Nucleus pulposus cells were inoculated in 96-well plates at a density of 1 × 10^4^ cells/well and incubated at 37 °C and 5% CO_2_ for 24 h. The cells were then treated with different concentrations of TBHP (0, 10, 50, 100, 150, 200, and 300 μM) for 6 h. After treatment, each well was supplemented with 10 μL CCK-8 reagent solution and further incubated under the same conditions for 1 h. Cell activity was assessed by measuring absorbance at 450 nm using a Bio-Tek, Winooski, VT, USA.

### 2.17. Slow Virus Transfection

When the density of NPCs in the culture flask was approximately 30%, 100 multiplicity infections were performed using the slow virus (GeneChem, Daejeon, Republic of Korea). The culture medium was changed 48 h after transfection. The expression of SOD2 was quantified using Western blotting.

### 2.18. Statistical Analysis

All results were based on at least 3 independent replicates. The technical repetition is only used for detecting internal variations. Quantitative data are expressed as standard deviation (SD) ± mean. Data analysis and statistical evaluation were performed using GraphPad Prism 8.0 Software (GraphPad Software, San Diego, CA, USA). For comparisons between two groups, Student’s *t*-test was used, while one-way analysis of variance (ANOVA) was used for comparisons involving more than two groups. Statistical significance was determined at a threshold of *p* < 0.05.

## 3. Results

### 3.1. Degenerated Grade IV NP Tissues in Humans Exhibit a Significant Reduction in Notochordal Cell Markers Compared to Normal Grade II NP Tissues

**Figure 1:**
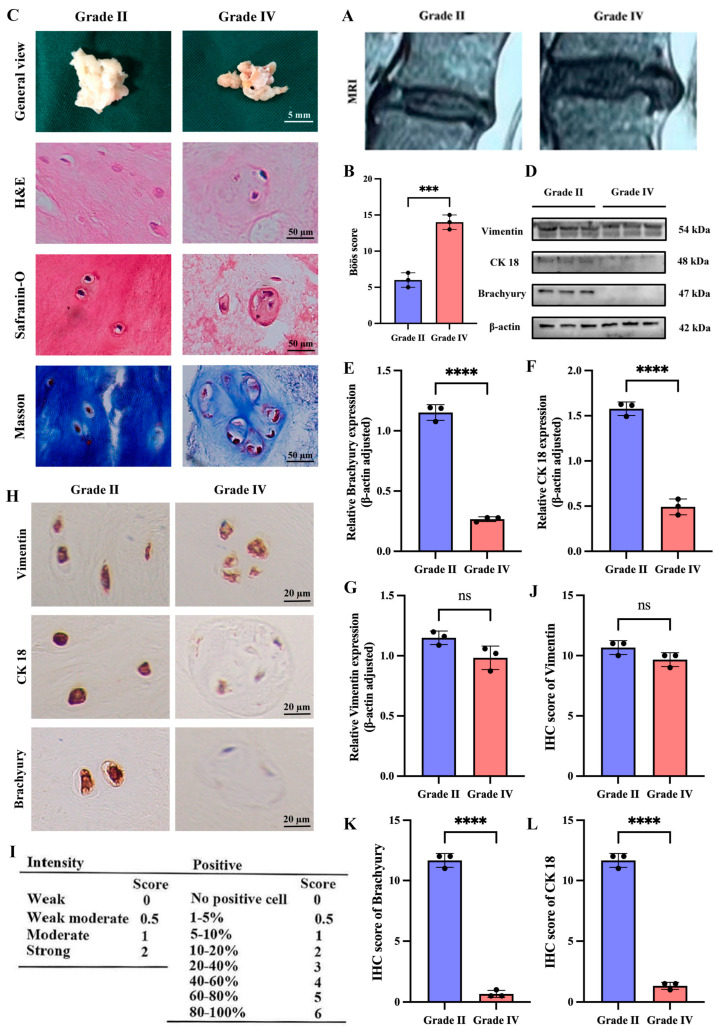
Degenerated grade IV NP tissues in humans exhibit a significant reduction in notochordal cell markers compared to normal grade II NP tissues. (**A**) MRI images of grade II and grade IV discs. (**B**) Böös score of IDD. (**C**) Macroscopic views of the nucleus pulposus tissue of grade II and IV discs and various pathological discolorations. (**D**–**G**) Western blotting and quantitative analysis of notochordal cell markers (CK 18, Brachyury) and the mesenchymal marker Vimentin in nucleus pulposus tissue of grade II and IV intervertebral discs. (**H**–**L**) Immunohistochemical staining and quantitative analysis of notochordal cell markers (CK 18, Brachyury) and the mesenchymal marker Vimentin in the nucleus Pulposus tissue from grade II and grade IV intervertebral discs. “ns” indicates no statistical difference between the two groups. *** *p* < 0.001, **** *p* < 0.0001.

To investigate the association between NCs and IDD, we obtained NP tissue from normal (Pfirrmann grade II) and degenerated (Pfirrmann grade IV) samples based on MRI based Pfirrmann method grading system. Grade II NP tissues have higher water content and appear as high signal intensity on MRI, while grade IV NP tissues have lower water content and show low signal intensity (Figure 1A). Subsequently, we performed the histological degeneration score (Figure 1B). Macroscopically, grade II NP tissues are gel-like, white, and elastic, whereas grade IV NP tissues are dark yellow and exhibit varying degrees of fibrosis (Figure 1C). Under H&E staining, grade II NP tissues contain small vacuolated cells with a uniformly dark ECM, while grade IV NP tissues contain large vacuolated cells that are highly aggregated with a uniformly light ECM (Figure 1C). Safranin O and Masson staining also revealed that the ECM of grade IV NP tissues had lower amounts of proteoglycans and collagen fibers compared to grade II NP tissues (Figure 1C). To confirm the presence of NCs in NP tissues with different degrees of degeneration, we performed Western blotting analysis for notochordal cell markers (CK18, Brachyury) and mesenchymal markers (Vimentin) in both grade II and Grade IV NP tissues. The results showed a significant reduction in notochordal cell markers in grade IV NP tissues compared to grade II NP tissues (Figure 1D–G). IHC analysis further confirmed these results (Figure 1H–L).

### 3.2. Reduction in Notochordal Cell Markers in NP Tissues of SD Rat IDD Model Induced by AF Needle Puncture

**Figure 2:**
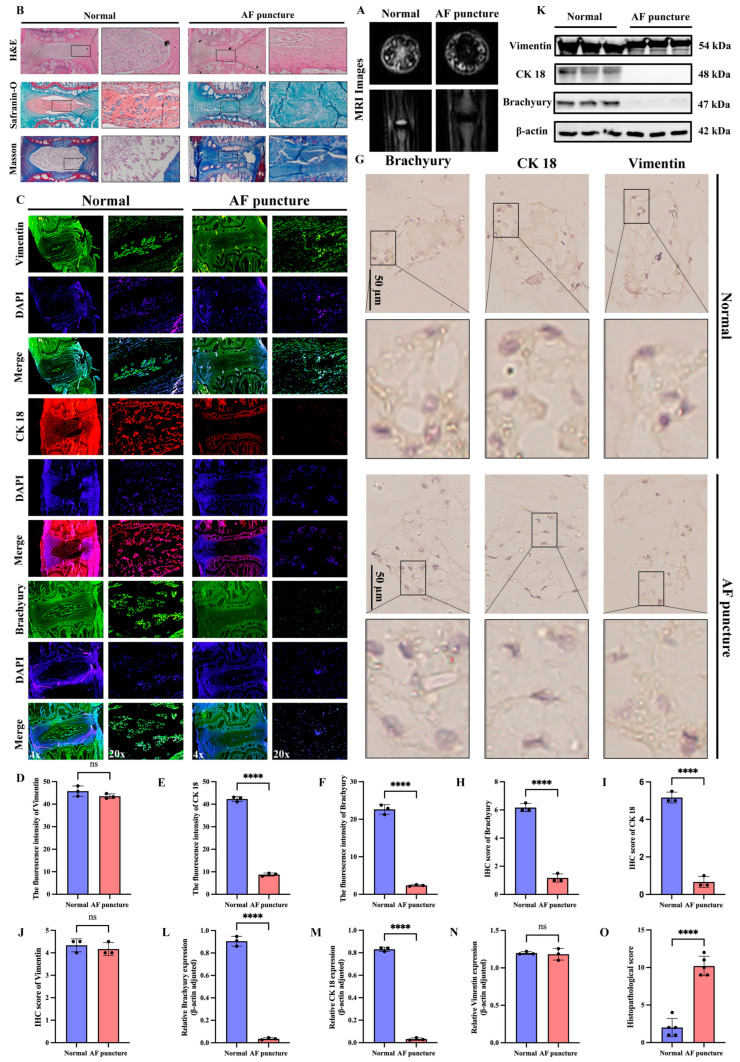
Reduction in notochordal cell markers in NP tissues of SD rat IDD model induced by AF needle puncture. (**A**) MRI images of the normal group and the AF acupuncture group. (**B**) Pathological staining of the intervertebral discs in SD rats of the normal group and the AF acupuncture group. (**C**–**F**) Immunofluorescence staining and quantitative analysis of notochordal cell markers (CK 18, Brachyury) and the mesenchymal marker Vimentin in intervertebral discs of SD rats of the normal group and the AF acupuncture group. (**G**–**J**) Immunohistochemical staining and quantitative analysis of notochordal cell markers (CK 18, Brachyury) and the mesenchymal marker Vimentin in intervertebral discs of SD rats of the normal group and the AF acupuncture group. (**K**–**N**) Western blotting and quantitative analysis of notochordal cell markers (CK 18, Brachyury) and the mesenchymal marker Vimentin in intervertebral discs of SD rats of the normal group and the AF acupuncture group. (**O**) Histopathological score was utilized to assess the extent of IDD in different groups. “ns” indicates no statistical difference between the two groups. **** *p* < 0.0001.

To further validate the accuracy and generalizability of the above experimental results, we used normal SD rats as controls and analyzed the IVDs 8 weeks after needle puncture of the AF. MRI evaluation revealed a significant loss of disc height, nuclear collapse, and a concomitant low T2 signal in the AF puncture group compared to the normal controls (Figure 2A). Histological staining revealed a significant reduction in IVD height in the AF puncture group, as well as disrupted AF structure, NP collapse, and fibrosis (Figure 2B). IF analysis showed that the AF puncture group had a significant reduction in notochordal cell markers (CK18, Brachyury) compared to the normal group, while the mesenchymal marker vimentin had no significant changes (Figure 2C–F). In addition, IHC analysis (Figure 2G–J) and Western blotting (Figure 2K–N) further confirmed the results of IF analysis. Histopathological score indicated that the AF puncture group had higher scores than the Normal group (Figure 2O).

### 3.3. Extraction and Identification of NCs and NPCs

**Figure 3:**
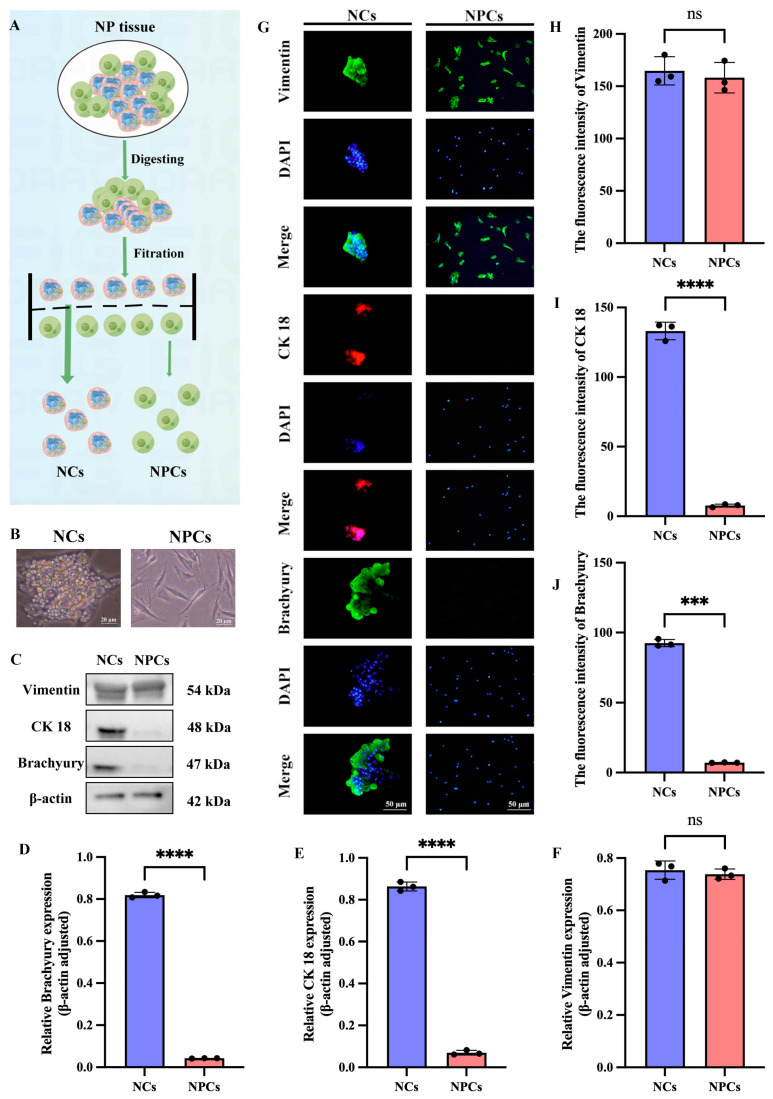
Extraction and identification of NCs and NPCs. (**A**) Schematic representation of NCs and NPCs. The dotted lines in the figure represent the filter screen, and the arrows indicate the process. (**B**) Microscopic observation of NCs and NPCs. (**C**–**F**) Western blotting results and quantitative analysis for the mesenchymal marker Vimentin and the notochord cell markers (CK 18, Brachyury) in NCs and NPCs. (**G**–**J**) Results and quantitative analysis of immunofluorescence staining for the mesenchymal marker Vimentin and the notochord cell markers (CK 18, Brachyury) in NCs and NPCs. “ns” indicates no statistical difference between the two groups. *** *p* < 0.001, **** *p* < 0.0001.

After establishing the connection between NCs and IDD, we aimed to develop new therapeutic strategies for IDD based on NCs. To achieve this, we extracted and identified NCs and NPCs from SD rats by following the procedure shown in Figure 3A. In contrast to the spindle-shaped NPCs, NCs were characterized by a spherical shape and the presence of vacuole-like structures (Figure 3B). To further confirm the identity of the two cell types, we then identified both using notochord cell markers (CK 18, Brachyury) and the mesenchymal marker vimentin. Western blotting results showed no significant statistical difference in the expression of vimentin between NCs and NPCs, while NCs had high expression of CK18 and Brachyury, which was absent in NPCs (Figure 3C–F). IF analysis of NCs and NPCs further confirmed these results (Figure 3G–J).

### 3.4. Identification of Differentially Expressed Genes (DEGs) in NCs and NPCs

**Figure 4:**
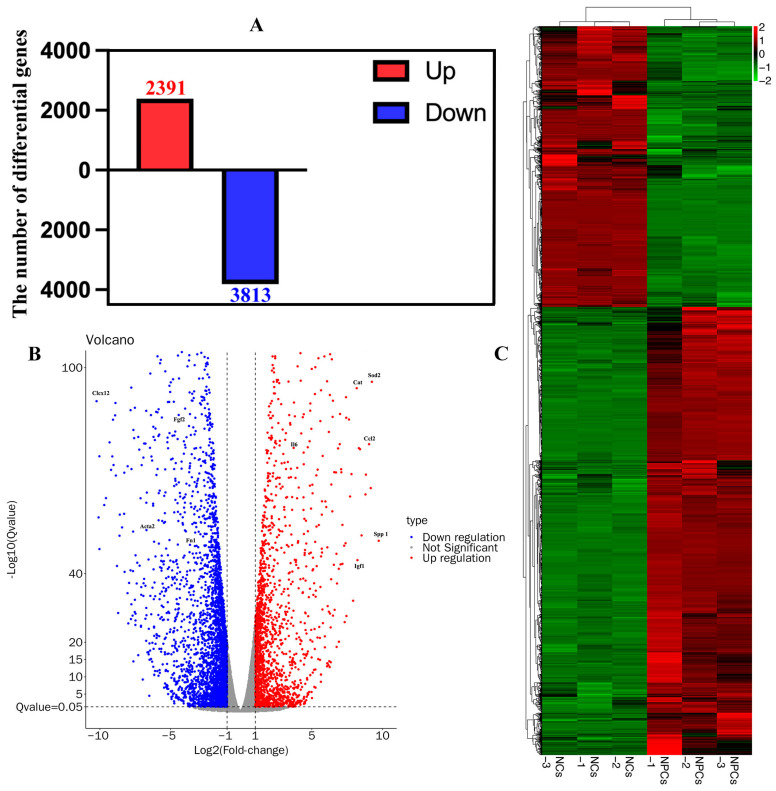
Identification of differentially expressed genes (DEGs) in NCs and NPCs. (**A**) A total of 6204 DEGs were identified in NCs, including 2391 upregulated DEGs and 3813 downregulated DEGs. (**B**) Volcano diagram of DEGs. Red indicates upregulated differential genes, while blue indicates downregulated differential genes. The vertical coordinate is −Log10(Qvalue). (**C**) Heatmap of DEGs. The clustered heatmap is based on gene expression levels. Red indicates highly expressed genes while green indicates low expressed genes. Each row represents the expression level of the same difference gene in different groups, and each column represents the expression level of all DEGs in different groups.

To identify the DEGs between NCs and NPCs, we first collected total RNA samples from three independent NC samples (named NCs-1, NCs-2, NCs-3) and three NPCs samples (named NPCs-1, NPCs-2, NPCs-3) using the MJzol Animal RNA Extraction Kit (MagBeads, MJ Biotechnology, Guangzhou, China) #T02096 (Reagent Lot Number: 2306010). We applied the spliced mapping algorithm of Hisat2 (version 2.0.4) [30] for genome mapping of the preprocessed reads (Supplementary Figure S1A). The distribution of genome coverage was visualized using a 1K window (Supplementary Figure S1B). We then performed correlation analysis of gene expression levels between samples and used principal component analysis (PCA) to evaluate relationships between samples (Supplementary Figure S1C,D). We performed differential gene analysis between samples using edgeR (version 4.0.0) [31], and after controlling for the False Discovery Rate (FDR) [32], DEGs were selected based on a Q-value ≤ 0.05 and |log2FC| ≥ 1. This filtering process identified a total of 2391 upregulated DEGs and 3813 downregulated DEGs when comparing the NCs group with the NPCs group (Figure 4A). We further visualized the DEGs using a volcano plot and a heatmap (Figure 4B,C).

### 3.5. GO Annotation and KEGG Enrichment Analysis of DEGs

**Figure 5:**
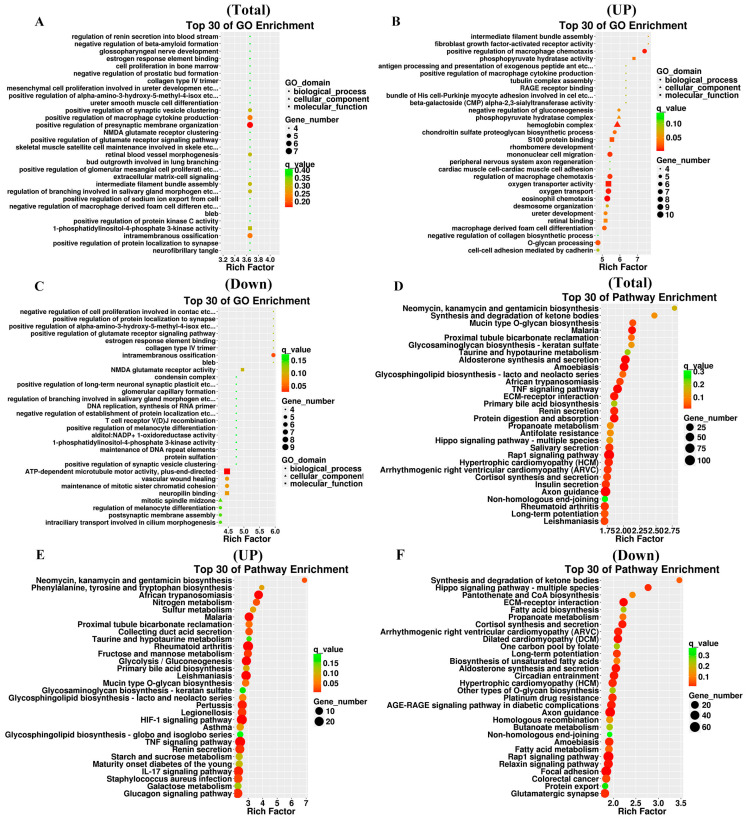
GO annotation and KEGG enrichment analysis of DEGs. (**A**) Scatter plot of the top 30 rich factor rankings in the GO analysis of DEGs. (**B**) Scatterplot of the top 30 rich factor rankings in the GO analysis of upregulated DEGs. (**C**) Scatter plot of the top 30 rich factor rankings in the GO analysis of downregulated DEGs. (**D**) Top 30 KEGG pathways sorted by enrichment level in KEGG analysis of DEGs. (**E**) Top 30 KEGG pathways sorted by enrichment level in KEGG analysis of upregulated DEGs. (**F**) Top 30 KEGG pathways sorted by enrichment level in KEGG analysis of downregulated DEGs. The rich factor is calculated as follows: (number of differential genes in a given GO term/total number of differential genes that can be mapped to the GO database)/(number of genes contained in a given GO term/total number Genes that can be assigned to the GO database). A larger rich factor indicates a higher level of enrichment. The q value is the *p* value after correction for multiple hypothesis testing, with smaller values indicating greater enrichment.

**Figure 6:**
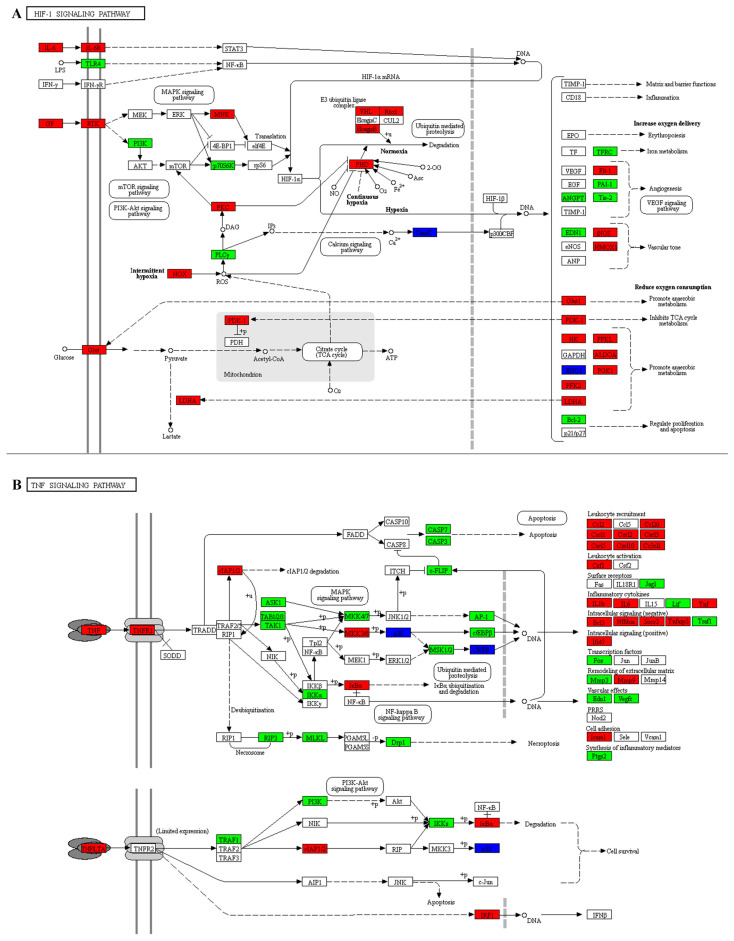
Differentially expressed proteins in the HIF-1 and TNF signaling pathways in KEGG. (**A**) The KEGG signaling pathway of HIF-1. (**B**) The KEGG signaling pathway of TNF. Differentially expressed proteins are shown in boxes and labeled with gene names. Solid line with arrow: Direct activation. Solid line with bar: Direct inhibition. Dashed line with arrow: Indirect activation/Transcription. Dashed line with bar: Indirect inhibition. Different colors represent protein expression levels, with red indicating upregulation, green indicating downregulation, and blue indicating the protein corresponds to multiple genes.

To investigate the function of DEGs, we performed GO and KEGG enrichment analysis on both upregulated and downregulated DEGs. The DEGs involved in biological processes are related to extracellular matrix signaling, regulation of ions inside and outside the cell, cell proliferation, regulation of cell aging, regulation of macrophage cytokine production, and regulation of associated signaling pathways (Figure 5A–C). The results of GO analysis suggest that the DEGs are involved in a variety of functions, including antioxidant activities. KEGG pathways include the TNF signaling pathway, Rap1 signaling pathway, regulation of glycolysis, fatty acid metabolism, cortisol synthesis and secretion, IL-17 signaling pathway, ECM-receptor interaction, Hippo signaling pathway, and HIF-1 signaling pathway (Figure 5D–F). To determine the role of DEGs in IDD, we conducted KEGG pathway analysis and found that the main pathways involved are the TNF and HIF-1 signaling pathways (Figure 6).

### 3.6. Screening of Target Genes Related to IDD

**Figure 7:**
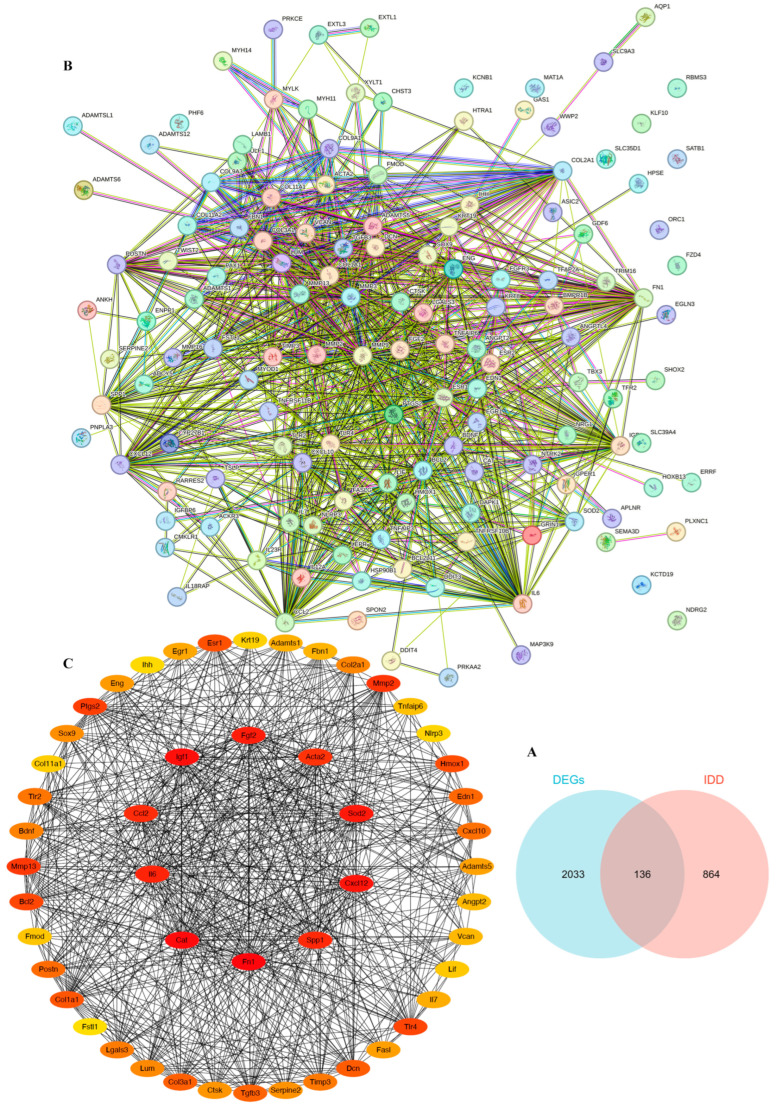
Screening of Target Genes Related to IDD. (**A**) Venn diagram of differential genes and IDD-related genes after screening. (**B**) A protein–protein interaction network of 136 genes. (**C**) The top 50 and 10 target genes associated with IDD were analyzed.

To screen for differentially expressed genes (DEGs) with potential therapeutic value for IDD, we selected 2169 DEGs with |log2FC| ≥ 2. We then intersected the 2169 DEGs with 1000 IDD-related genes from https://www.genecards.org (accessed on 22 February 2024), resulting in 136 DEGs (Figure 7A). Next, we performed a protein–protein interaction (PPI) network analysis of these DEGs using the STRING database at https://cn.string-db.org (accessed on 10 May 2024) (Figure 7B). Finally, we predicted the top 50 target genes related to IDD, with the top 10 target genes being *Sod2*, *Cxcl12*, *Spp1*, *Fn1*, *Cat*, *Il6*, *Ccl2*, *Igf1*, *Fgf2*, and *Acta2* (Figure 7C).

### 3.7. Differential Expression of SOD2 and CAT in Human Normal Grade II NP Tissues and Degenerated Grade IV NP Tissues

**Figure 8:**
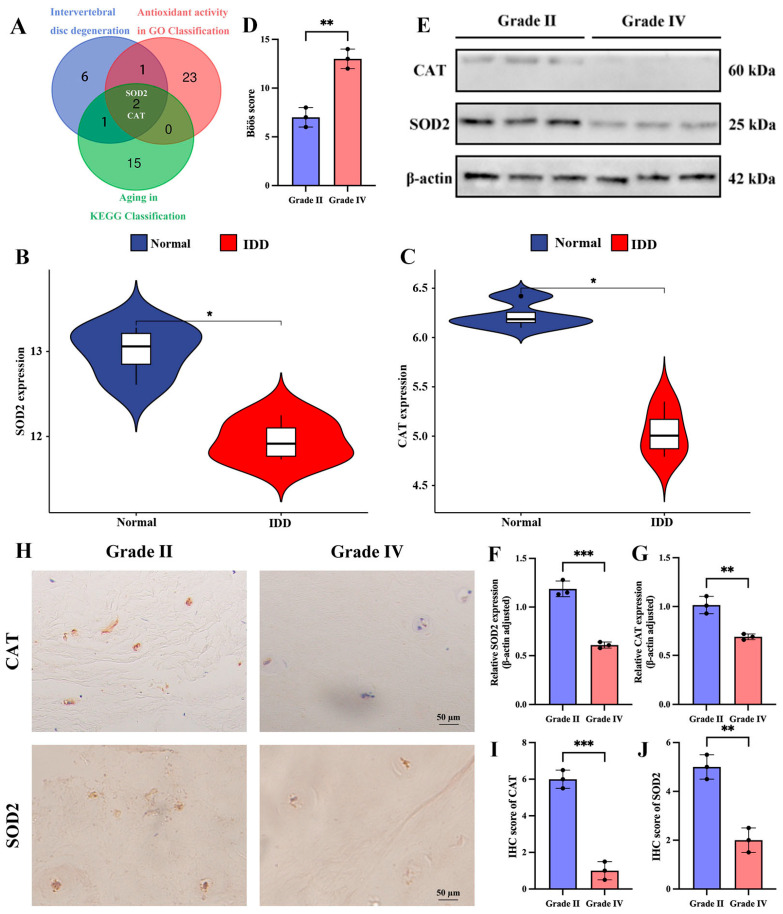
Differential expression of SOD2 and CAT in human normal grade II NP tissues and degenerated grade IV NP tissues. (**A**) Screening of SOD2 and CAT. (**B**,**C**) Violin plots of SOD2 and CAT in normal and degenerated intervertebral discs of humans. (**D**) Böös score of IDD. (**E**–**G**) Western blotting and quantitative analysis of SOD2 and CAT in grade II and grade IV disc nucleus pulposus tissues. (**H**–**J**) Immunohistochemical staining and quantitative analysis of SOD2 and CAT in grade II and grade IV disc nucleus pulposus tissues. * *p* < 0.05, ** *p* < 0.01, *** *p* < 0.001.

Candidate molecular targets for IDD therapy were identified through an integrated bioinformatic approach. This involved intersecting antioxidant genes from GO classification, senescence-related genes from KEGG pathways, and the top 10 IDD-associated key genes, which pinpointed SOD2 and CAT (Figure 8A). To further strengthen the connection of our candidates to IDD, we performed an in silico validation using a publicly available dataset (GSE147383) containing transcriptomes of human NP tissues from non-degenerated and degenerated discs. Reassuringly, both SOD2 and CAT mRNA levels were significantly downregulated in the degenerated group compared to the normal group (*p* < 0.05) (Figure 8B,C). This independent analysis confirms that the expression of these genes, which we first identified as being enriched in NCs, is indeed suppressed in the context of human IDD, strongly supporting their relevance as therapeutic targets. Subsequently, we performed the histological degeneration score (Figure 8D). To investigate the relationship between SOD2 and CAT and IDD, we collected human relatively normal Grade II NP tissue and severely degenerated Grade IV NP tissue and performed pathological staining identification. The results of Western blotting analysis showed that SOD2 and CAT were highly expressed in grade II NP tissues and weakly expressed in grade IV NP tissues (Figure 8E–G). Furthermore, our IHC analysis confirmed the results of Western blotting (Figure 8H–J).

### 3.8. Decreased Expression of SOD2 and CAT in the IDD Model of SD Rats with AF Puncture

**Figure 9:**
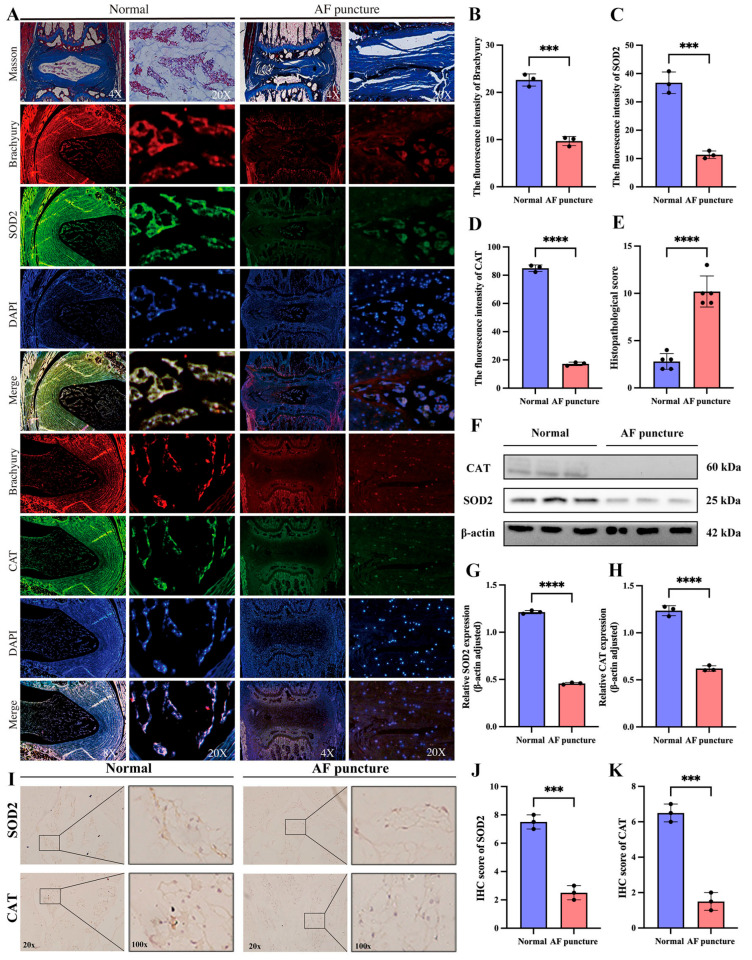
Decreased expression of SOD2 and CAT in the IDD model of SD rats with AF puncture. (**A**) Masson staining and tissue immunofluorescence of SOD2 and CAT in SD rats of the normal group and the AF acupuncture group. (**B**–**D**) Quantitative analysis of tissue immunofluorescence of SOD2 and CAT in SD rats of normal group and AF acupuncture group. (**E**) Histopathological score was utilized to assess the extent of IDD in different groups. (**F**–**H**) Western blotting and quantitative analysis of SOD2 and CAT in SD rats of the normal group and the AF acupuncture group. (**I**–**K**) Immunohistochemical staining and quantitative analysis of SOD2 and CAT in SD rats of the normal group and the AF acupuncture group. *** *p* < 0.001, **** *p* < 0.0001.

To further verify whether the relationship between SOD2 and CAT and IDD is universal, we also examined the IVD of normal SD rats as a control after 8 weeks of AF puncture. The Masson staining results showed that we successfully established the IDD model (Figure 9A). To directly determine whether the observed downregulation of SOD2/CAT occurs in NCs or NPCs, we performed multiplex IF co-staining on intervertebral disc tissues obtained from both control and AF puncture-induced injury groups in rats. Tissue sections were simultaneously incubated with antibodies against the notochordal cell-specific marker Brachyury (red), together with antibodies targeting either SOD2 or CAT (green), and counterstained with DAPI (blue) for nuclear visualization. In the AF Puncture (IDD) group, we observed a dual phenomenon: (i) a dramatic reduction in the number of Brachyury + NCs, and (ii) a clear attenuation of the SOD2 and CAT fluorescence intensity within the remaining NCs as well as in the surrounding Brachyury-negative NPC population.

The results of tissue immunofluorescence showed that the expression of SOD2 and CAT was significantly reduced in the AF puncture group compared to the normal group (Figure 9A–D). Histopathological score indicated that the AF puncture group had higher scores than the Normal group (Figure 9E). Western blotting analysis (Figure 9F–H) and IHC analysis (Figure 9I–K) further confirmed the tissue immunofluorescence results.

### 3.9. In a Model of Oxidative Stress and Cellular Senescence Induced by TBHP in NPCs from Humans and SD Rats, the Expression of SOD2 and CAT Was Significantly Reduced

**Figure 10:**
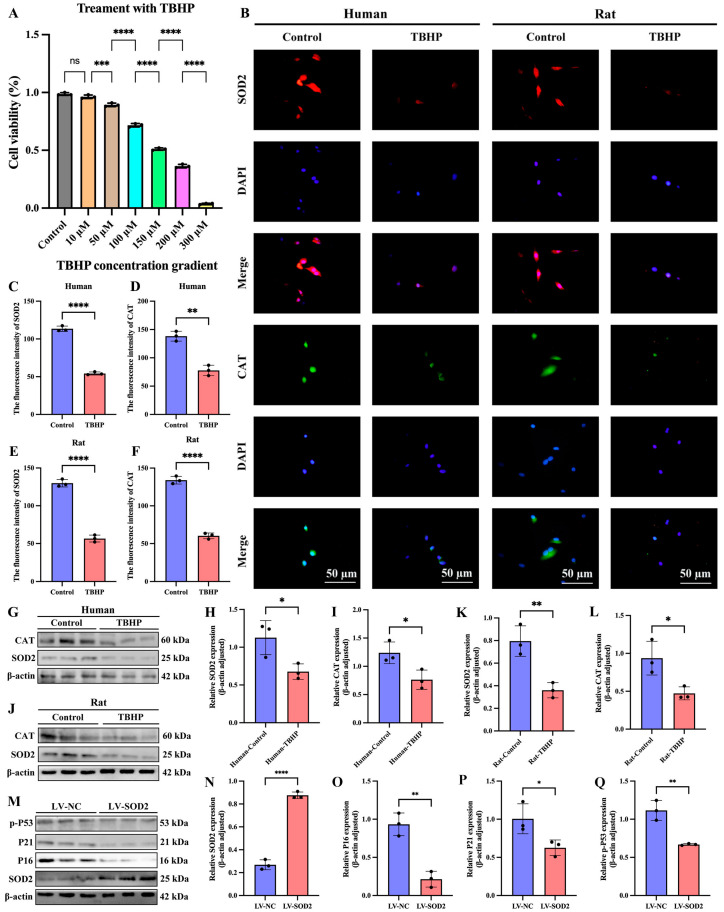
In a model of oxidative stress and cellular senescence induced by TBHP in NPCs from humans and SD rats, the expression of SOD2 and CAT was significantly reduced; LV-SOD2 can inhibit the senescence of NPCs. (**A**) The cell counting kit experiment is used to determine the cell viability of NPCs treated with different concentrations of TBHP. (**B**–**F**) Results of immunofluorescence staining and quantitative analysis of two nucleus pulposus cell lines from humans and SD rats. (**G**–**L**) Western blotting and quantitative analysis of two nucleus pulposus cell lines from humans and SD rats. (**M**–**Q**) The expression of aging markers in the LV-SOD2 NP cell line. “ns” indicates no statistical difference between the two groups. * *p* < 0.05, ** *p* < 0.01, *** *p* < 0.001, **** *p* < 0.0001.

To further validate the bioinformatic screening results in Figure 8A, specifically whether SOD2 and CAT affect oxidative stress and cellular senescence, we constructed models of oxidative stress and cellular senescence in human and SD rat nucleus pulposus cell lines using tert-butyl hydroperoxide (TBHP). First, we treated NPCs with different concentrations of TBHP (0, 10, 50, 100, 150, 200, and 300 μM) for 6 h. We then assessed cell viability using the Cell Counting Kit-8 (CCK-8) assay (Figure 10A). Based on the cell viability results, we chose 150 μM TBHP as the optimal concentration to induce oxidative stress and cellular senescence in NPCs for subsequent experiments. IF results of NPCs in both humans and SD rats showed that the expression of SOD2 and CAT was significantly reduced in the TBHP-induced oxidative stress and senescence groups compared to the control groups (Figure 10B–F). Western blotting further confirmed the results of IF analysis (Figure 10G–L).

### 3.10. LV-SOD2 Can Inhibit the Senescence of NPCs

We used lentiviral vectors to transfect human NPCs to establish a superoxide dismutase 2 overexpression (LV-SOD2) cell line. We used Western blotting to detect the expression levels of senescence-related proteins in the LV-SOD2 NP cell lines. The results showed that overexpression of SOD2 could reduce the expression of senescence markers P16, P21 and p-P53 (Figure 10M–Q). These results indicate that SOD2 can delay the aging of NPCs.

## 4. Discussion

IDD, a common age-related degenerative disease of the spine, is characterized by the aging and shrinkage of NPCs [33,34,35]. More importantly, the decline in NPCs contributes to the development and progression of IDD [36]. However, current treatments for IDD cannot prevent chronic progression [37,38]. Therefore, maintaining IVD homeostasis by promoting NPC proliferation and inhibiting NPC senescence would be an ideal therapeutic strategy for IDD [39]. Recent studies suggest that NPCs in the NP are mainly derived from a large number of NCs [40]. This could ensure a steady supply of NPCs from the source and provide a potential therapeutic strategy for IDD based on NCs.

In certain vertebrates such as mice, rabbits, cows, and dogs, NCs remain in NP tissue for most of their lifespan, and these species rarely exhibit significant IDD during their lifetime [20,41]. In contrast, in humans, NCs begin to decline after the second decade of life, coinciding with the early onset of IDD [42]. Fortunately, our studies suggest that although NCs are significantly reduced in severely degenerated NP tissues compared to relatively normal NP tissues, a small number of notochordal cell markers can still be detected. Previous studies have shown that NCs and their secretions play a role in disc regeneration by promoting NPCs proliferation, inhibiting NPCs apoptosis, suppressing ECM degradation, reducing inflammation, inhibiting angiogenesis and differentiation surrounding mesenchymal stem cells in NPCs [12,17,43,44,45]. Therefore, targeting the remaining NCs in degenerated IVDs for therapeutic interventions in IDD seems to be a promising approach.

To further investigate potential molecular targets for NC-based IDD therapy, we performed transcriptomic analysis and experimental validation on NCs and NPCs from SD rats. In this study, we conducted GO and KEGG analyzes to investigate key molecular functions, biological processes, and signaling pathways. GO enrichment analysis revealed that DEGs related to antioxidant activity, response to external stimuli, cell proliferation, metabolism, ECM cell signaling and regulation of macrophage cytokine production were significantly enriched. The dynamic balance between ECM synthesis and degradation is crucial for maintaining healthy NP tissues, and any disruption of this balance can lead to IDD. Recent studies have shown that in IDD patients, the concentrations of proteoglycans and collagen type II in NP tissues are significantly reduced, while the concentrations of collagen type I are increased, resulting in decreased water retention, mechanical dysfunction, and impaired ECM response leads to mechanical stress [46,47]. In terms of immune regulation, NPCs can modulate cytokine production in macrophages. This regulatory effect is likely mediated through the release of intercellular signaling molecules, ultimately influencing the local inflammatory state and healing progression.

Analysis of the KEGG signaling pathway revealed that DEGs are associated with biological processes such as cellular aging, movement, and growth and death. Cellular senescence is a major risk factor for the progression of IDD as it leads to the accumulation of senescent NPCs in the IVD [48]. During IDD, the rate of NPCs senescence increases significantly and these cells secrete various metabolic factors, such as pro-inflammatory cytokines, matrix-degrading enzymes, growth factors and chemokines, which alter the ECM [49,50,51]. Enriched KEGG signaling pathways include TNF signaling pathway, Rap1 signaling pathway, HIF-1 signaling pathway and Hippo signaling pathway. The cytokines IL-1β and TNF-α are important pro-inflammatory mediators with strong inflammatory activities and promote the secretion of various pro-inflammatory mediators. These cytokines are considered key factors in IDD and LBP. IL-1β and TNF-α are upregulated in degenerative IVDs and are closely related to pathological processes in IDD, including inflammation, matrix degradation, cellular senescence, autophagy, apoptosis, pyroptosis and proliferation [52]. Therefore, anti-IL-1β and anti-TNF-α therapies may have the potential to alleviate IDD and LBP. Hypoxia-inducible factor 1 (HIF-1) plays an important role in energy metabolism and ECM synthesis [53]. Furthermore, HIF-1 can activate macroautophagy/autophagy through various pathways [54,55]. These properties enable HIF-1 to regulate the homeostasis and stem cell function of several endogenous stem cells [56]. In advanced IDD, neovascularization increases the oxygen concentration in the IVD [57], which accelerates the oxygen-dependent degradation of HIF-1 [58]. Recent studies have shown that depletion of HIF-1 further disrupts the homeostasis of nucleus pulposus stem cells (NPSCs). However, maintaining a hypoxic environment may help IVD cells adapt to the degenerative environment of advanced IDD [33]. Therefore, maintaining adequate HIF-1 levels in NCs and NPSCs may be an effective strategy to slow the progression of IDD.

Cellular senescence and oxidative stress are prominent features of NPCs in IDD. Based on the above analysis, we identified two molecules associated with cellular senescence and oxidative stress: SOD2 and CAT. We found that the expression of SOD2 and CAT was decreased in IDD tissues from humans and SD rats. Using a TBHP-induced cellular senescence and oxidative stress model in both human and SD rat cell lines, we confirmed the association of SOD2 and CAT with oxidative stress and senescence. Superoxide dismutase 2 (SOD2) and catalase (CAT) are the main antioxidant enzymes in cells. SOD2 converts superoxide radicals into hydrogen peroxide, which is then further broken down into water and oxygen by CAT, effectively scavenging reactive oxygen species (ROS) and protecting cells from oxidative damage [59]. Our investigations found that the expression levels of SOD2 and CAT were significantly reduced in disc degeneration, which may contribute to NPC dysfunction and disc degeneration. Therefore, increasing the activity of these antioxidant enzymes could open a new way to treat intervertebral disc degeneration. However, the exact mechanisms of SOD2 and CAT in IDD and their relationship to each other are still poorly understood. While existing research suggests a close connection between oxidative stress and disc cell health, there are inconsistencies between studies. Future research should focus on improving the methodological consistency and rigor of study design to better compare and analyze the results of different studies.

It is important to address a key consideration regarding our experimental design. The transcriptomic analysis compared two distinct cell types—NCs and NPCs—from healthy rats. Therefore, the identified DEGs primarily reflect differences in cellular identity and baseline function, rather than changes directly caused by the degeneration process itself. We cannot conclusively state that these DEGs are de facto drivers of IDD based solely on this comparison. However, our strategy was predicated on the hypothesis that the unique molecular signature of resilient NCs harbors clues for mitigating degeneration. The subsequent validation of the top candidates, SOD2 and CAT, demonstrating their significant downregulation in independent human degenerated tissues and experimental IDD models, provides strong circumstantial evidence that the loss of these NC-enriched, protective factors is a critical event in IDD pathogenesis.

Furthermore, our sequencing results provide clues for the identification of specific biomarkers for NCs. Although we previously summarized NCs markers, no specific markers for NCs have been identified to date [12]. In our mRNA sequencing results, FXYD6, RAB38, and GPC3 were highly expressed in NCs and not in NPCs, suggesting that these molecules may serve as NC-specific markers, which requires further validation in future studies.

Although our study confirmed the relationship between NCs and IDD and identified two potential therapeutic targets based on mRNA sequencing of NCs and NPCs, there are several limitations. First, due to technical limitations and suboptimal RNA sample concentration, we only selected NCs and NPCs from SD rats for sequencing and did not include RNA samples from human NCs and NPCs, which provides a direction for future research. Second, the results of the bioinformatics analysis were based on predefined criteria and changing the thresholds could affect the final results. Third, although the needle injection-induced rat IDD model is a useful research tool, rats are rodents and their IVDs differ in their functional and biomechanical properties from those of humans, limiting direct comparison with human pathology. The consistency and reproducibility of surgical procedures is challenging, and this model cannot fully simulate the complex mechanisms of human IDD. Future studies need to consider using animal models that are closer to humans to gain more detailed insights into the mechanisms of human IDD. Furthermore, the human control NP tissues were obtained from adolescent idiopathic scoliosis patients, as acquiring truly healthy human disc tissue from age-matched individuals is ethically and practically challenging. While we confirmed the histological integrity of these control tissues, we cannot entirely rule out potential influences of the scoliotic mechanical environment on the molecular profile of the NP cells. Future studies utilizing post-mortem specimens from healthy donors could help validate our findings.

## 5. Conclusions

Compared to normal NP tissue, the expression of notochordal cell markers was markedly reduced in degenerated tissue, although a low level remained readily detectable. SOD2 and CAT may serve as potential therapeutic targets for IDD by alleviating oxidative stress and cellular senescence in NPCs.

## Data Availability

The analyzed datasets generated during the present study are available from the corresponding author (E-mail: ery_kangxw@lzu.edu.cn) on reasonable request.

## Supplementary Materials

The following supporting information can be downloaded at: https://www.mdpi.com/article/10.3390/cimb47121001/s1.

## Abbreviations

The following abbreviations are used in this manuscript:

IDD	Intervertebral disc degeneration
NCs	Notochordal cells
NP	Nucleus pulposus
IHC	Immunohistochemistry
NPCs	Nucleus pulposus cells
GO	Gene Ontology
KEGG	Kyoto Encyclopedia of Genes and Genomes
DEGs	Differentially expressed genes
TBHP	Tert-butyl hydroperoxide
AF	Annulus fibrosus
CEP	Cartilaginous endplates
ECM	Extracellular matrix
LBP	Low back pain
NSAIDs	Non-steroidal anti-inflammatory drugs
MSC	Mesenchymal stem cells
NPPCs	Nucleus pulposus progenitor cells
