# Supplementary material for: Comprehensive Transcriptomic Analysis and Experimental Validation of Notochordal Cells and Nucleus Pulposus Cells: Uncovering Novel Therapeutic Targets for Intervertebral Disc Degeneration
Source: Curr Issues Mol Biol. 2025 Nov 28;47(12):1001. doi: 10.3390/cimb47121001 (PMC12732074; doi:10.3390/cimb47121001)
Supplement: Supplementary file 1 [file cimb-47-01001-s001.zip › Supplementary materials-Table 1.pdf]

Table 1: Quality control results of total RNA samples from NCs and NPCs

Sample Name	Con.	Vol.	Total (μg)	A260/ A280	2100 Result		Result
					RIN	28S/18S	
NCs-1	191.3	45	8.61	1.93	10.0	1.9	A1
NCs-2	183.8	45	8.27	1.93	10.0	2.0	A1
NCs-3	169.3	45	7.62	1.92	10.0	2.1	A1
NPCs-1	267	45	12.02	1.92	10.0	2.2	A1
NPCs-2	199.2	45	8.96	1.92	10.0	2.1	A1
NPCs-3	213.1	45	9.59	1.92	10.0	2.0	A1

Note: A1 indicates that both RNA integrity and total amount meet the library construction requirements, and the experiment can continue.
