# Supplementary figures and images for: Comprehensive Transcriptomic Analysis and Experimental Validation of Notochordal Cells and Nucleus Pulposus Cells: Uncovering Novel Therapeutic Targets for Intervertebral Disc Degeneration
Source: Curr Issues Mol Biol. 2025 Nov 28;47(12):1001. doi: 10.3390/cimb47121001 (PMC12732074; doi:10.3390/cimb47121001)

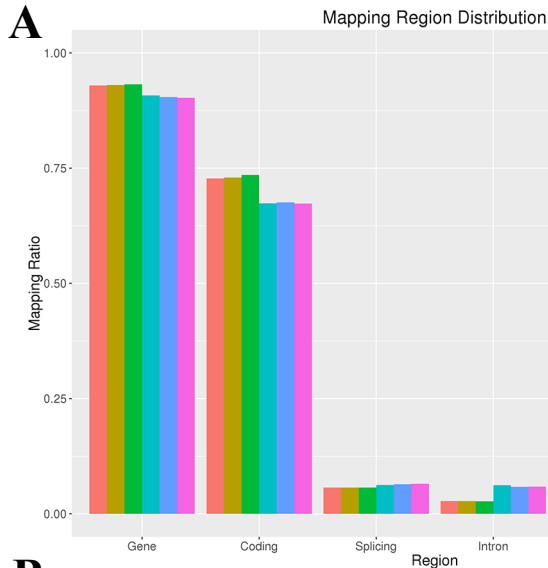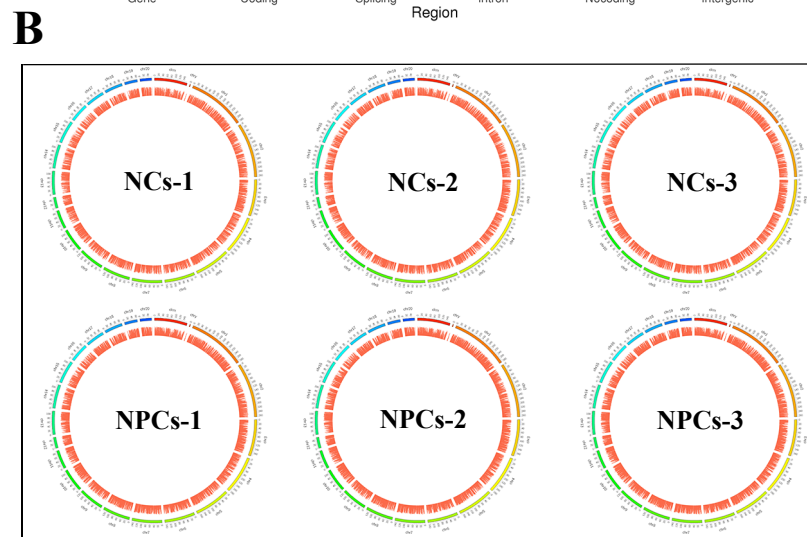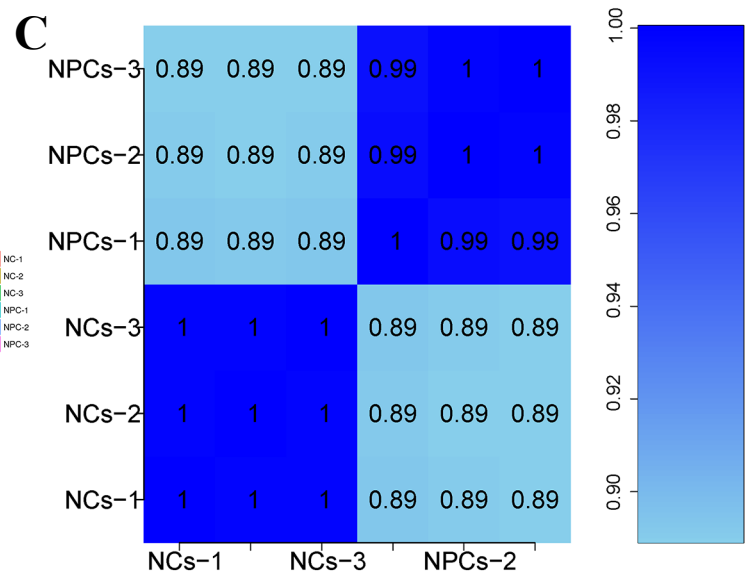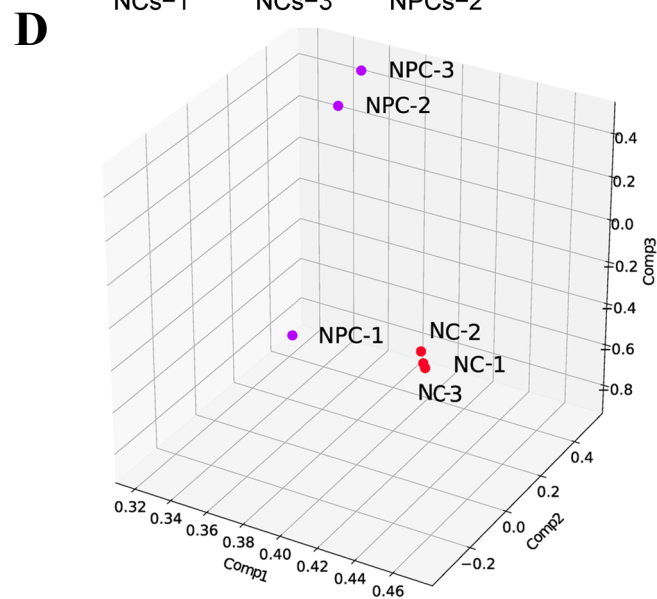

Supplement: Supplementary file 1 [file cimb-47-01001-s001.zip › Supplementary materials Figure S1.pdf]
